# Genetic evidence for a causative effect of airflow obstruction on left ventricular filling: a Mendelian randomisation study

**DOI:** 10.1186/s12931-021-01795-9

**Published:** 2021-07-07

**Authors:** Lars Harbaum, Jan K. Hennigs, Marcel Simon, Tim Oqueka, Henrik Watz, Hans Klose

**Affiliations:** 1 Abteilung für Pneumologie, Centrum für Pulmonal Arterielle Hypertonie Hamburg (CPAHH), Universitätsklinikum Hamburg-Eppendorf, Hamburg, Germany; 2 Pneumologische Forschungsinstitut an der LungenClinic Grosshansdorf, Airway Research Center North (ARCN), German Center for Lung Research (DZL), Grosshansdorf, Germany

## Abstract

**Background:**

Observational studies on the general population have suggested that airflow obstruction associates with left ventricular (LV) filling. To limit the influence of environmental risk factors/exposures, we used a Mendelian randomisation (MR) approach based on common genetic variations and tested whether a causative relation between airflow obstruction and LV filling can be detected.

**Methods:**

We used summary statistics from large genome-wide association studies (GWAS) on the ratio of forced expiratory volume in 1 s to forced vital capacity (FEV1/FVC) measured by spirometry and the LV end-diastolic volume (LVEDV) as assessed by cardiac magnetic resonance imaging. The primary MR was based on an inverse variance weighted regression. Various complementary MR methods and subsets of the instrument variables were used to assess the plausibility of the findings.

**Results:**

We obtained consistent evidence in our primary MR analysis and subsequent sensitivity analyses that reducing airflow obstruction leads to increased inflow to the LV (odds ratio [OR] from inverse variance weighted regression 1.05, 95% confidence interval [CI] 1.01–1.09, P = 0.0172). Sensitivity analyses indicated a certain extent of negative horizontal pleiotropy and the estimate from biased-corrected MR-Egger was adjusted upward (OR 1.2, 95% CI 1.09–1.31, P < 0.001). Prioritisation of single genetic variants revealed rs995758, rs2070600 and rs7733410 as major contributors to the MR result.

**Conclusion:**

Our findings indicate a causal relationship between airflow obstruction and LV filling in the general population providing genetic context to observational associations. The results suggest that targeting (even subclinical) airflow obstruction can lead to direct cardiac improvements, demonstrated by an increase in LVEDV. Functional annotation of single genetic variants contributing most to the causal effect estimate could help to prioritise biological underpinnings.

**Supplementary Information:**

The online version contains supplementary material available at 10.1186/s12931-021-01795-9.

## Introduction

Observational studies based on the general population have consistently demonstrated that increasing airflow obstruction can be associated with reduced left ventricular (LV) filling [1–3]. The relationship between the ratio of forced expiratory volume in 1 s to forced vital capacity (FEV1/FVC) as a measure of airflow obstruction in pulmonary function tests and LV end-diastolic volumes (LVEDV) was nearly linear across the spectrum of lung function in the Multi-Ethnic Study of Atherosclerosis (MESA) Lung Study [1]. Similarly, a decline in FEV1/FVC ratio was associated with underfilling of the left heart in the longitudinal Coronary Artery Risk Development in Young Adults (CARDIA) study [2]. An increase of airflow obstruction has also been associated with cardiovascular outcomes in the general population. In the Copenhagen City Heart Study (CCHS) the FEV1/FVC ratio was related to the risk of cardiovascular death independent of traditional cardiovascular risk factors [4]. These observations indicate that subclinical airflow obstruction may be linked with hemodynamic changes in the pulmonary circulation leading to reduced inflow to the LV.

Pulmonary and cardiac function variables are complex traits and large, population-based genome-wide association studies (GWAS) have provided multiple independent associations between genetic variation (mostly single-nucleotide polymorphism [SNP]) and quantitative measurements across the genome [5–8]. The principle of testing for an association of the same genetic variant with different traits allows to conclude on causality based on shared genetic risk. A Mendelian randomisation (MR) framework can help to provide such evidence and can be viewed as a meta-analysis of the causal estimates from each independent genetic variant [9]. Notably, since genetic variants are randomly allocated at conception, a MR approach limits the influence of environmental risk factors/exposures.

Using MR, we tested whether airflow obstruction may be casually linked to LV filling in large general population datasets.

## Methods

### Selection of instruments for Mendelian randomisation

In a two-sample Mendelian randomisation (MR) approach the instrument variables (IV) are developed from the association between common genetic variation and a risk factor or exposure (here FEV1/FVC) [10]. The outcome variable contains associations of these genetic variants with a second trait (here LVEDV) in an independent sample [10]. We used publicly available summary statistics (effect alleles, estimates and their standard errors) for SNPs that associated with FEV1/FVC in a GWAS meta-analysis of the United Kingdom (UK) Biobank and SpiroMeta populations comprising up to 404,128 individuals to generate the IV (up to 321,047 individuals from the UK Biobank and 79,055 from the SpiroMeta consortium) [7]. We selected SNPs that associated with FEV1/FVC at genome-wide level of significance (P < 5 × 10^–9^) [11] in the GWAS meta-analysis [7] and excluded palindromic variants (A/T or C/G) with intermediate allele frequencies (minor allele frequency > 45%) to ensure that effects of variants for the exposure and outcome traits can be harmonized to the same allele. We retained common (minor allele frequency [MAF] > 5%) and uncorrelated SNPs (linkage disequilibrium [LD] r^2^ < 0.001 within a ± 10 Mb window in the European 1000 Genomes phase 3 reference panel using PLINK 1.9 software), which were also available for the outcome. The outcome variable was obtained from a GWAS on LVEDV as measured by cardiac magnetic resonance imaging (MRI) in 36,042 individuals from the UK Biobank [5].

We tested for association of the IV with covariates querying all variants or proxies in high LD (r^2^ > 0.8) for association with disease endpoints or complex traits using a curated database of human genotype–phenotype associations (PhenoScanner V2) [12]. We did not consider association with pulmonary function test traits and intermediate phenotypes such as protein, metabolite or expression levels. To assess the influence on the causal estimate, we excluded variants that associated with one or more traits at genome-wide significance (P < 5 × 10^–9^) followed by retesting.

To ensure that the overlap of individuals from the UK Biobank in the exposure and outcome cohorts did not overly influence our results (e.g., because of ‘winner’s curse’ or overfitting) [13], we used a subset the IV including only SNPs that showed an association (P < 1 × 10^–3^) in the SpiroMeta consortium population [14] and retested the causal estimate.

### Reverse Mendelian randomisation

Applying identical data processing for a reverse MR analysis, we considered common and uncorrelated SNPs identified in the GWAS on LVEDV at genome-wide significance (P < 5 × 10^–9^) as IV (exposure) [5]. The corresponding outcome variable was obtained from the GWAS meta-analysis of the UK Biobank and SpiroMeta populations on FEV1/FVC [7, 14]. Again, palindromic SNPs were excluded and only common and uncorrelated SNPs were retained.

### Statistical analyses

The statistical approach of the MR strategy followed recent recommendations [15]. Primary MR analysis was performed using the inverse variance weighted (MR-IVW) regression method under a multiplicative random-effects model using TwoSampleMR v0.5.4 software package in R-3.6.1 [16]. The MR-IVW method assumes no horizontal pleiotropy, where genetic variants independently associate with traits other than the ones under investigation, but will be unbiased if there is balanced pleiotropy.

In subsequent sensitivity analyses, we applied MR based on Egger regression (MR-Egger) [17] and weighted median (MR-WM) [18]. MR-Egger regression provides valid causal estimate when the InSIDE (Instrument Strength Independent of Direct Effect) assumption holds, even when all SNPs are invalid [17]. We used the adopted I^2^ statistic (referred to as I^2^_GX_) to assess the NOME (no measurement error) assumption [19]. I^2^_GX_ provides an assessment of the degree of regression dilution due to uncertainty in the SNP-exposure associations and was proposed as a measure of instrument strength for the MR-Egger method [19]. MR-Egger intercept and funnel plots plotting the strength of variant associations with the exposure (as the inverse standard error) against the individual IV estimates were generated to assess whether pleiotropy is balanced or directional [17]. The MR-WM method has been proposed to complement MR-Egger in a sensitivity analyses as it does not to rely on the InSIDE assumption [18]. MR-WM provides valid causal estimates when 50% of the information is contributed from valid SNPs [18].

We additionally applied the MR-PRESSO (Mendelian Randomisation Pleiotropy RESidual Sum and Outlier) method to our MR model to identify and remove horizontal pleiotropic outlier variants followed by retesting for heterogeneity [20].

To assess the effects of individual SNPs, we performed single SNP MR analyses on each SNP individually using the simple ratio estimate (dividing the effect of the IV on the outcome by the effect of the IV on the exposure) and leave-one-out MR analyses, in which the causal estimate is retested using the MR-IVW regression method after sequentially omitting one SNP.

Findings at P < 0.05 were considered statistically significant.

## Results

### Mendelian randomisation of FEV1/FVC on LVEDV

**Fig. 1:**
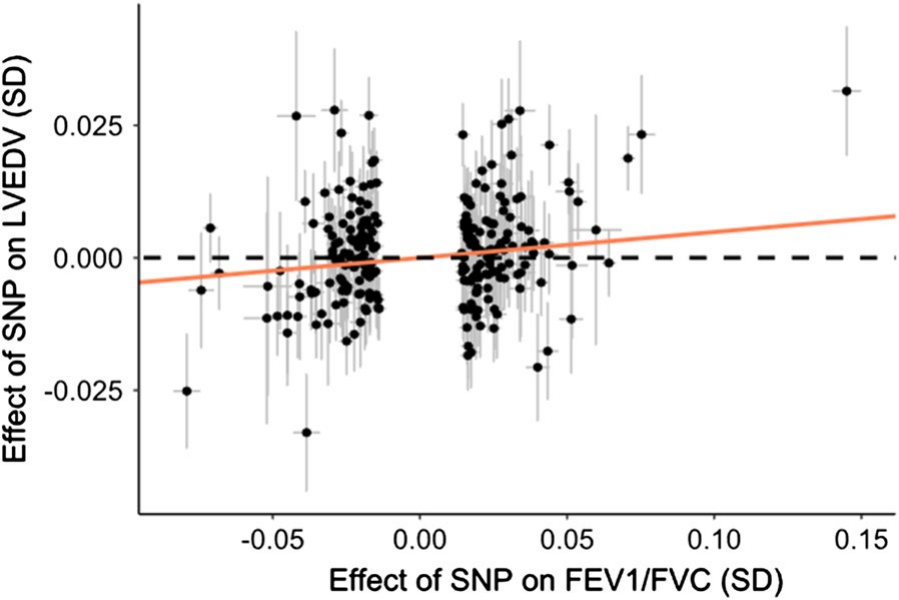
Scatter plot of the effect estimates and standard error from 253 genetic variants on both FEV1/FVC and LVEDV. The orange line shows the overall causal effect from the inverse variance weighted regression

Using 253 SNPs independently associated with FEV1/FVC in a GWAS meta-analysis of UK Biobank and SpiroMeta consortium populations, we found genetic evidence for a causative effect of FEV1/FVC on LVEDV in our primary MR-IVW analysis with an odds ratio (OR) of 1.05 per increase of 1 standard deviation in FEV1/FVC (95% confidence interval [CI] 1.01–1.09, P = 0.017; Fig. 1 and Table 1). Directionality was consistent between Mendelian randomisation analysis and pervious observational data [1–3]. Full details on the variant filtering steps and IV can be found in the online supplement (Additional file 1: Fig. S1 and Additional file 2: Table S1).

**Table 1:** Results of Mendelian randomisation (MR) analyses of FEV1/FVC on LVEDV

Instrument variables (IV)		MR method	SNPs	Odds ratio	95% confidence interval		Estimate	Standard error	P-value
					Lower	Upper			
Full (primary IV)		MR-IVW	253	1.05	1.01	1.09	0.05	0.02	0.017
		MR-WM	253	1.06	1.01	1.12	0.06	0.03	0.026
		MR-Egger	253	1.20	1.09	1.31	0.18	0.05	< 0.001
		MR-PRESSO	250	1.05	1.02	1.10	0.05	0.02	0.007
Subset	No association to potential covariates	MR-IVW	146	1.07	1.01	1.13	0.06	0.03	0.019
		MR-WM	146	1.05	0.98	1.14	0.05	0.04	0.185
		MR-Egger	146	1.19	1.01	1.40	0.18	0.08	0.038
	Association with FEV1/FVC in the SpiroMeta consortium population	MR-IVW	73	1.06	1.00	1.12	0.06	0.03	0.043
		MR-WM	73	1.05	0.97	1.13	0.05	0.04	0.214
		MR-Egger	73	1.12	0.98	1.28	0.12	0.07	0.091
		MR-PRESSO	72	1.06	1.01	1.12	0.06	0.03	0.021

The direction of effect estimates was consistent in subsequent sensitivity analyses (Table 1). The I^2^_GX_ for the SNP-exposure associations was 0.99 indicating that attenuation due to NOME violation was small. A funnel plot displayed no asymmetry indicative for directional pleiotropy (Additional file 1: Fig. S2). The intercept from Egger-regression, however, suggested a certain extent of negative directional pleiotropy (Additional file 2: Table S2). Consequently, the bias-corrected MR-Egger estimate was adjusted upward (OR 1.2, 95% CI 1.09–1.31, P < 0.001; Table 1). The MR-WM provided a similar causal estimate as compared to the MR-IVW (OR 1.06, 95% CI 1.01–1.12, P = 0.028; Table 1). There was evidence of heterogeneity in the SNP effect (Cochrane’s Q 402) and MR-PRESSO identified three influential outliers (rs4353138, rs7253302 and rs75928020) with an outlier test P = 0.025. With removal of these outliers, the causal effect estimate remained similar (OR 1.05, 95% CI 1.02–1.10, P = 0.007; distortion test P = 0.84; Table 1). We next performed leave-one-out MR-IVW analyses (excluding one variant at a time) and observed lower confidence interval ranging from 1 to 1.01 and upper from 1.08 to 1.1 (Additional file 2: Table S3).

To test for associations of the IV with covariates, we found that 107/253 variants associate with 291 different disease endpoints or other complex traits (Additional file 2: Table S4). With removal of these variants, a constant direction of effect estimates was observed in the primary MR-IVW (OR 1.09, 95% CI 1.03–1.16, P = 0.003) and subsequent sensitivity MR methods (Table 1). A funnel plot and the intercept from Egger-regression did not indicate directional pleiotropy, and MR-PRESSO did not identified outlier (Additional file 1: Fig. S2 and Table 2).

**Table 2:** Results of 19 SNPs that shared the direction of the overall Mendelian randomisation (MR) effect and were significant in single SNP MR analyses of FEV1/FVC on LVEDV

SNP	Minor allele	MAF (1000 Genomes Phase 3)	MAF (Exposure)	Relation to nearest gene	Single SNP MR analyses						Percentage decrease of overall effect in leave-one-out MR-IVW
					Odds ratio	95% confidence interval		Estimate	Standard error	P-value	
						Lower	Upper				
rs995758	T	0.30	0.40	Downstream, *HHIP*	1.3	1.1	1.54	0.27	0.09	0.002	16.7
rs2070600	T	0.07	0.06	Missense, *AGER*	1.24	1.05	1.46	0.22	0.08	0.01	13.4
rs7733410	A	0.45	0.44	Intron, *HTR4*	1.33	1.05	1.67	0.28	0.12	0.016	9.3
rs1192415	G	0.22	0.19	Upstream, *TGFBR3*	1.62	1.16	2.27	0.48	0.17	0.005	8.1
rs113315602	C	0.03	0.09	Downstream, *HLA-DRB1*	1.37	1.05	1.79	0.32	0.14	0.02	7.9
rs840467	T	0.12	0.19	Downstream, *LOX*	2.39	1.46	3.91	0.87	0.25	< 0.001	7
rs281861363	T	0.04	0.09	Intron, *HLA-DQB1*	1.36	1.02	1.82	0.31	0.15	0.037	6.5
rs13332450	C	0.23	0.93	Downstream, *FAM65A*	2.35	1.34	4.13	0.86	0.29	0.003	5.3
rs10059996	G	0.40	0.36	Upstream, *FGF18*	1.43	1.01	2.03	0.36	0.18	0.045	5.3
rs4353138	T	0.37	0.46	Downstream, *IPP*	4.91	2.23	10.84	1.59	0.4	< 0.001	5.1
rs9874847	T	0.17	0.14	Intron, *WNT7A*	2.48	1.37	4.5	0.91	0.3	0.003	5
rs7118465	C	0.43	0.32	Intron, *NTM*	1.88	1.14	3.09	0.63	0.25	0.012	4.9
rs201191	A	0.40	0.49	Intron, *AL445623.2* (lncRNA)	1.82	1.08	3.06	0.6	0.27	0.025	4.2
rs10460533	A	0.23	0.29	Intron, *EML4*	2.18	1.19	3.98	0.78	0.31	0.011	4.2
rs12134019	T	0.05	0.14	Intron, *HMCN1*	1.87	1.08	3.21	0.62	0.28	0.024	4
rs2285261	A	0.49	0.38	Intron, *MICU3*	2.09	1.12	3.89	0.74	0.32	0.021	3.7
rs6433079	C	0.34	0.76	Intron, *CERS6*	1.91	1.04	3.5	0.65	0.31	0.036	3.4
rs4721457	C	0.11	0.15	Upstream, *AC006041.1* (lncRNA)	2.06	1.06	4.01	0.72	0.34	0.034	3.1
rs62283793	T	0.07	0.05	Intron, *FNDC3B*	2.26	1.06	4.84	0.82	0.39	0.035	2.8

To test for an overestimation of the causal effect due to the overlap of individuals from the UK Biobank in the exposure and outcome cohorts, we selected 73/253 variants from the IV that showed association in SpiroMeta consortium population [14] and observed a constant direction of effect estimates in the primary MR-IVW (OR 1.06, 95% CI 1–1.12, P = 0.043) and in subsequent sensitivity analyses (Table 1). A funnel plot and the intercept of Egger-regression did not indicate directional pleiotropy (Additional file 1: Fig. S1 and Table 2). MR-PRESSO, however, identified rs75928020 as horizontal pleiotropic outlier variants (outlier test P = 0.0073). With removal, the causal effect remained similar (OR 1.06, 95% CI 1.01–1.12, P = 0.021; distortion test P = 0.88; Table 1).

To assess the relevance of individual SNPs, we performed single SNP MR analyses on each SNP individually and found that 141/253 SNPs shared the direction of the overall MR effect, from which 19/141 were also nominally significant in single SNP MR analyses (P < 0.05; Table 2 and Additional file 2: Table S3). In leave-one-out MR-IVW analyses on these 19 SNPs, the causal effect estimate was reduced between 3 to 16% compared to the overall MR-IVW result (Table 2 and Additional file 2: Table S3).

### Reverse Mendelian randomisation

To further understand a source of observational associations between FEV1/FVC and LVEDV in the general population, we performed the reversed MR experiment and used LVEDV-associated SNPs as instrument variable (exposure) to explore the relationship with FEV1/FVC (outcome). Using 13 SNPs independently associated with LVEDV, we found no evidence of an effect of LVEDV on FEV1/FVC using the primary MR-IVW (OR 1.02, 95% CI 0.94–1.1, P = 0.62).

## Discussion

Using a MR approach, we identified a causal relationship between FEV1/FVC and LVEDV. Our data provide genetic context to observational associations that earlier studies have reported [1–3], indicating a relationship between airflow obstruction and LV filling in the general population. Importantly, we found no evidence for a reverse-causation phenomenon. To date, we are not aware of a MR study that directly evaluated the effect of airflow obstruction on LV filling.

Several mechanisms of impaired LV filling with increasing airflow obstruction can be considered. Loss of pulmonary vasculature can occur even without manifest lung disease and the total volume of the pulmonary vasculature was associated with reduced cardiac filling [21]. Furthermore, pulmonary hyperinflation, which is particularly evident in patients with chronic obstructive pulmonary disease (COPD), has been suggested to decrease intrathoracic blood flow and reduce LV preload [22–24]. Interestingly, randomised and placebo-controlled trials have shown that in patients with COPD and hyperinflation bronchodilators increased LVEDV with an inverse correlation between treatment-associated change in residual volume and LVEDV [25, 26]. Using dual bronchodilator (indacaterol and glycopyrronium) achieved an increase of LVEDV by approximate 10% [25]. Alveolar hypoxia, in part a consequence of ventilatory changes, can also occur under physiological conditions and triggers hypoxic pulmonary vasoconstriction [27]. If sustained this mechanism contributes to volume and pressure overload of the right ventricle (RV), which in the context of diastolic ventricular interdependence decreases LV volumes [28]. An increased pulmonary vascular resistance and RV afterload is well established in patients with COPD and is in general dependent on the severity of the disease [29].

Assessing the individual SNPs in relation to the overall estimate could elucidate molecular underpinnings of the observed causal relationship between FEV1/FVC and LVEDV. Nineteen SNPs exhibited a significant effect in single SNP MR and shared the effect direction of the overall result. The top three SNPs with major contribution to the overall effect were rs995758 (chr4:145,478,201-C-T), rs2070600 (chr6:32,151,443-C-T) and rs7733410 (chr5:147,856,522-G-A). When leaving one of these three SNPs out, the effect of the overall MR-IVW was reduced by 17%, 13% and 9%, respectively. These loci have not only been linked to pulmonary function parameters such as FEV1/FVC [7], but also to COPD phenotypes including emphysema [30] and airway remodeling [31]. Rs995758 is located intergenic and the nearest gene, *HHIP,* encodes for the hedgehog interacting protein (HHIP). Mice with heterozygous *HHIP* knock-out (*HHIP*^+/−^) spontaneously develop airway remodeling with increased airway smooth muscle mass and increased airway resistance [32]. Rs2070600 is a missense variant in the *AGER* gene encoding for the type I receptor for advanced glycation end-products (RAGE). This variant is predicted to be tolerable (raw combined annotation dependent depletion [CADD] score of 3.5) but influences protein abundance [33, 34]. Protein expression of RAGE in airway cells was also implicated in sensitization after pulmonary allergen exposure and with immune regulatory function at a later stage of asthmatic disease [35]. Interestingly, variation at rs2070600 associated with outcomes in heart failure patients but not with risk of heart failure in a Chinese population [36]. The soluble form of RAGE (sRAGE) can be measured in the circulation and lower levels could serve as a biomarker for airflow obstruction [34], while levels seem to be elevated in patients with heart failure, indicating that interpretation of this biomarker in the context of cardiopulmonary interaction remains unclear [37]. The variant rs7733410 is located intronic to the *HTR4* gene encoding the 5-hydroxytryptamine (5-HT)/serotonin receptor 4 (HTR4). Mice deficient for this protein (*HTR4*^*−/−*^*)* were found to have increased airway resistance and exhibit increased methacholine-induced airway hyperresponsiveness [38]. Since HTR4 is temporally expressed in developing human lungs, it could potentially be implicated in lung development and physiological cardiopulmonary interaction [39].

We acknowledge as limitation that we cannot fully exclude residual confounders. Common diseases or risk factors potentially affecting FEV1/FVC and/or LVEDV could be present in a subgroup of individuals from the general population and could provoke a systematic bias, particularly in settings when pleiotropic effects of multiple genetic variants act through the same confounder (i.e., violated InSIDE assumption). By excluding all variants with known GWAS signals in a sensitvity analysis, we sought to control for this scenario. In addition, overlapping datasets for the exposure and outcome variable could cause potential overfitting. In a sensitivity analysis, we therefore selected variants associated with FEV1/FVC in the smaller subpopulation of the GWAS meta-analysis (SpiroMeta consortium population) [14].

## Conclusion

In summary, our present study provides genetic evidence for a causal link between airflow obstruction and LV filling by investigating the largest-to-date GWAS on FEV1/FVC and LVEDV in the general population. Our results suggest that even subclinical reduction in airflow impairs LV function, and that targeting airflow obstruction can lead to direct cardiac improvements, demonstrated by an increase in LVEDV. Functional annotation of single SNPs contributing most to the causal effect estimate could help to prioritise biological pathways for biomarker-discovery and therapeutic intervention.

## Supplementary Information

**Additional file 1: Fig. S1.** Variants filtering steps applied. Blue boxes indicate the set of IV used to estimate the causal effect. **Fig. S2.** Funnel plots on the three different IVs used to estimate the causal effect. Point estimate from IVW and MR-Egger displayed.

**Additional file 2: Table S1.** Detailed information on the IV used in the MR analyses. **Table S2.** Intercept from Egger-regression analyses. **Table S3.** Results from single SNP MR based on the ratio estimate and from leave-one-out MR based on the IVW method. **Table S4.** Association of the IV with potential covariates retrieved from a curated database of human genotype-phenotype associations. P-value of association displayed. 

## Data Availability

The datasets and bioinformatical tools supporting the conclusions of this article are publicly available [5, 7, 16].

## Abbreviations

CARDIA: Coronary Artery Risk Development in Young Adults Study
CCHS: Copenhagen City Heart Study
CI: Confidence interval
COPD: Chronic obstructive pulmonary disease
FEV1: Forced expiratory volume in 1 s
FVC: Forced vital capacity
GWAS: Genome-wide association studies
HHIP: Hedgehog interacting protein
HTR4: 5-hydroxytryptamine receptor 4
InSIDE: Instrument Strength Independent of Direct Effect
IV: Instrument variable(s)
IVW: Inverse variance weighted
LD: Linkage disequilibrium
LV: Left ventricular
LVEDV: LV end-diastolic volume
MAF: Minor allele frequency
MESA: Multi-Ethnic Study of Atherosclerosis Lung Study
MR: Mendelian randomisation
NOME: No measurement error
OR: Odds ratio
PRESSO: Pleiotropy RESidual Sum and Outlier
RAGE: Receptor for advanced glycation end-products
RV: Right ventricle
SNP: Single-nucleotide polymorphism
UK: United Kingdom
WM: Weighted median
