# Supplementary material for: Genetic evidence for a causative effect of airflow obstruction on left ventricular filling: a Mendelian randomisation study
Source: Respir Res. 2021 Jul 7;22:199. doi: 10.1186/s12931-021-01795-9 (PMC8261939; doi:10.1186/s12931-021-01795-9)
Supplement: Supplementary file 1 — Additional file 1: Fig. S1. Variants filtering steps applied. Blue boxes indicate the set of IV used to estimate the causal effect. Fig. S2. Funnel plots on the three different IVs used to estimate the causal effect. Point estimate from IVW and MR-Egger displayed. [file 12931_2021_1795_MOESM1_ESM.pdf]

Genetic evidence for a causative effect of airflow obstruction on left ventricular filling: a Mendelian randomisation study

Supplementary Figures

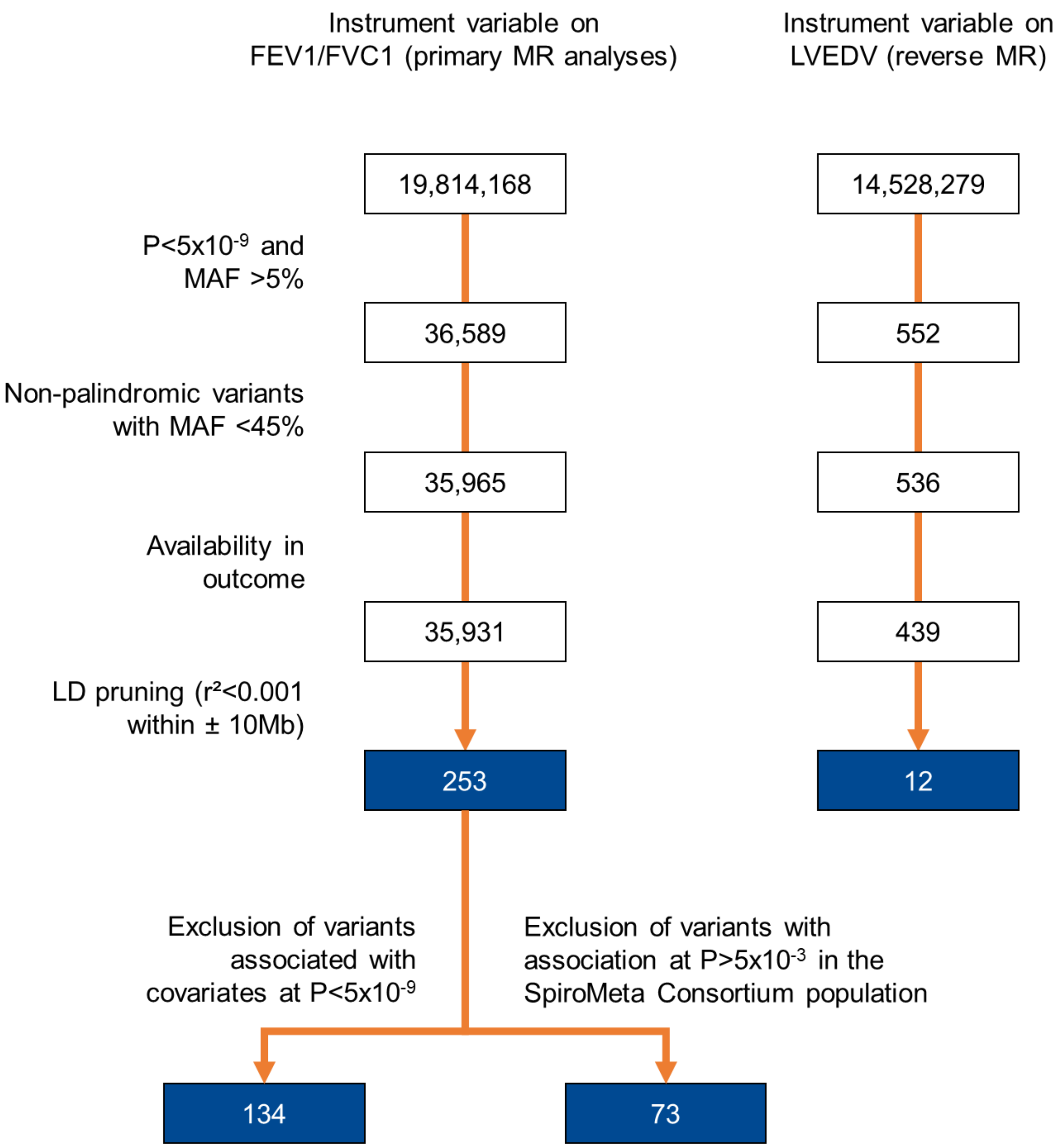

Supplementary Figure 1: Variants filtering steps applied. Blue boxes indicate the set of IV used to estimate the causal effect.

Full IV (253 variants)

Subset of IV (no association with potential covariates; 146 variants)

Subset of IV (association with FEV1/FVC in the SpiroMeta consortium population; 73 variants)

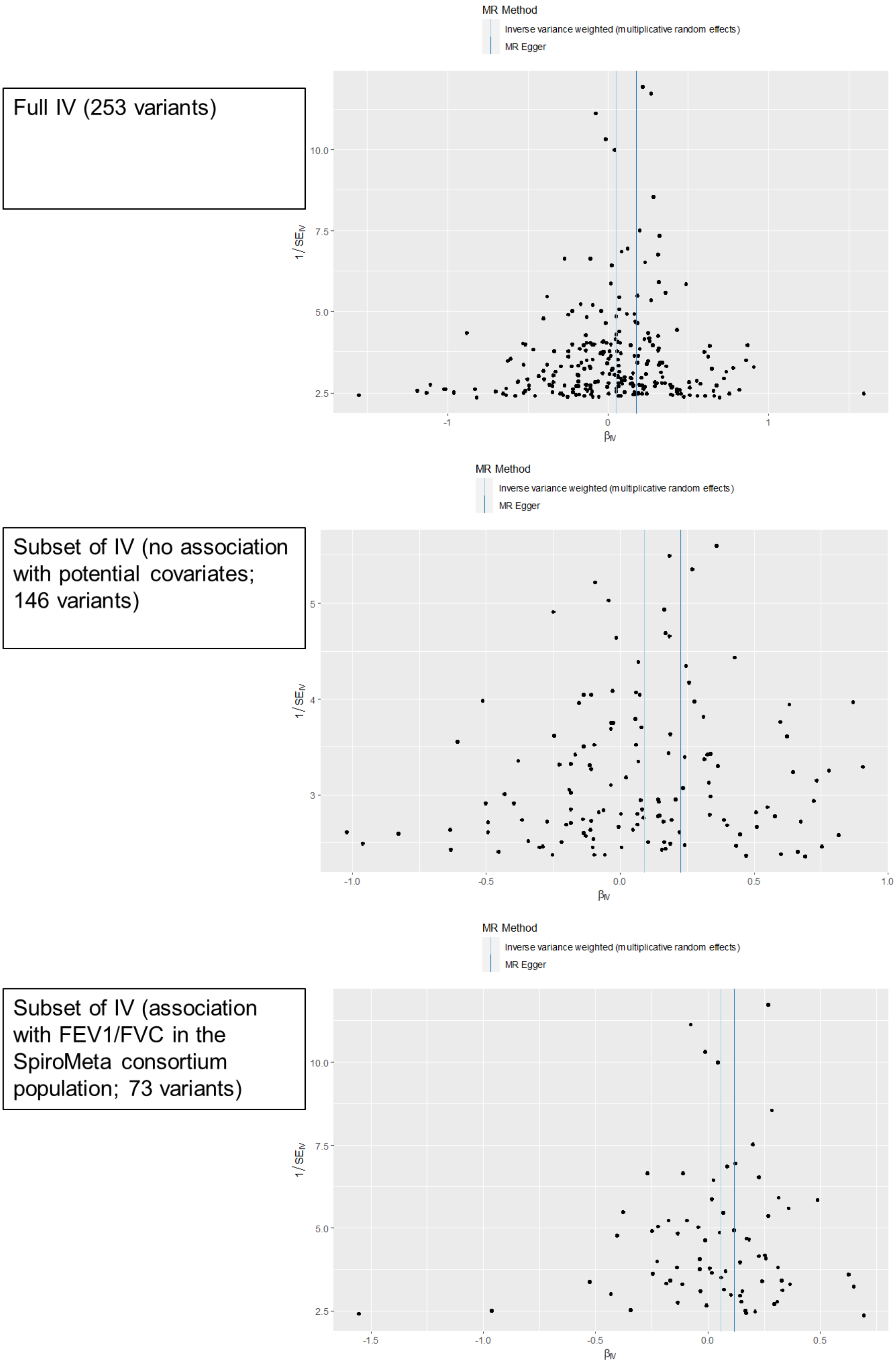

Supplementary Figure 2: Funnel plots on the three different IVs used to estimate the causal effect**.** Point estimate from IVW and MR-Egger displayed.
